# Hospital and Institutional News

**Published:** 1912-03-23

**Authors:** 


### HOSPITAL AND INSTITUTIONAL NEWS.

### NEW NAMES ON THE TUBERCULOSIS COMMITTEE.

The Chancellor of the Exchequer has added the following names to the Committee which is to report upon the considerations of general policy in dealing with tuberculosis in the United Kingdom: Mr. Arthur Henderson, M.P., and Mr. F. J. Willis, one of the Assistant Secretaries of the Local Government Board, who has been acting as Secretary to the Committee. Mr. O. B. Clarke, barrister-at-law, has been appointed Secretary of the Committee. From this, apparently, it is safe to say that Mr. R. Clement Lucas' suggestion for the addition of a surgical authority has not yet been carried out. As he pointed out at the time, the present Committee is apparently only to deal with one form of a very general infecting disease. An explanation of this limitation is necessary.

### ROYAL VISIT TO GUY'S HOSPITAL.

The King and Queen visited Guy's Hospital last Saturday, arriving there about 3.15, intimation of their intention having been received only the previous afternoon. Lord Goschen, the treasurer, and Sir Cooper Perry, M.D., the superintendent and physician to the Skin Department, received their Majesties, who remained in the hospital for about an hour and a half. The King and Queen first inspected the Finsen light and Röntgen ray departments, where they spent some time, and then made their way to the new theatres. After inspecting these their Majesties passed on to the surgical and medical wards, frequently stopping to speak to the patients, some of whom were brought to their special attention. Then, as if to mark his first visit to Guy's since his accession, the King went into the hospital grounds, thus reversing the order of progress which he made when he visited the London on the first public appearance of his reign, and there again conversed with several of the patients who were out of doors. "Hullo!" said one little boy in response to a question as to his ailment, an unceremonial reply which created a good deal of amusement. His Majesty's last words, "It is about two years since I last visited your hospital, but I hope it will not be so long before I come again," were among the most encouraging tokens of the King's interest, and assure the whole hospital world, through the compliment paid to Guy's, of the active sympathy which his Majesty continues to take in everything concerned with the voluntary system.

### HOSPITAL MANAGEMENT FORTY YEARS AGO AND NOW.

Up to forty years ago the management of the voluntary hospitals and many kindred institutions, though nominally by committee, actually resulted in the control being vested in a relatively few hands. The old idea of glorifying benevolence by recording on huge and often hideous boards the names of benefactors of considerable sums, the patronage which resulted from the ticket system of admission and other causes, combined to promote the absorption of authority, so far as the management was concerned, in a small number of people often connected by family ties. A good side was undoubtedly that those who took the responsibility often gave largely in money and much personal service to the particular institutions with which they associated themselves. The evils were a narrow policy and the almost necessarily personal considerations which governed much of the procedure in regard to such institutions. We refer our readers to an article in the current issue on "Management by Committee" which affords evidence that ancient methods may still be met with in voluntary hospitals throughout the country.

### THE LORD MAYOR ON DOCTORS IN PUBLIC LIFE.

Sir Thomas Crosby, M.D., speaking at the festival dinner of the Irish Medical Schools last week, took as his text the place of medical men in public life, and urged the profession to take a more active part in public affairs. "The medical profession," he said, "has never taken its proper stand in municipal or parliamentary life, although members of the profession are, as a rule, the best-educated men throughout the kingdom." As we pointed out, the influence the profession can exert in these spheres is well illustrated by the result of the South Manchester election, which was a medical victory and nothing else. Sir Thomas Crosby, whose election to the post of chief magistrate gives the practical example to his precept, then assured the meeting that it was for the sake of his profession, as a matter of professional principle in fact, that he had determined to be the first medical man to become Lord Mayor of London. We have already had proofs that the Mansion House, which has done so much in the past for the cause of voluntary charity, will make this year especially memorable.

### A MAYOR AS HOSPITAL SECRETARY.

It is not so uncommon as it used to be for the honorary secretary of a voluntary hospital to be elected mayor of his town; but though we recall a case or two, the latest example is worth adding to the list. Mr. H. O. Williams, honorary secretary of the Luton Bute Hospital, a post which he has held for almost twenty years, is now mayor also, and special mention was recently made of his services to the hospital, which seemed to show, as his approachability was emphasised, that institutional work need not suffer from combination with public duties. The ways in which a hospital can benefit from possessing a secretary who is also mayor are obvious, but both offices are becoming so busy nowadays that it is only in the less populous districts that the combination has been tried. There is, however, no doubt that the cause of voluntary hospitals gains from the fact of public officials, who do much for local charities, having, as in the case of Sir Thomas Crosby, M.D., Lord Mayor of London, and of Mr. Williams, Mayor of Luton, intimate personal knowledge of institutional life and work.

### PORTSMOUTH PLEADS "NOT GUILTY."

A fortnight ago some criticisms were offered in these columns upon certain features in the management of the Royal Portsmouth Hospital. In the *Hampshire Telegraph* an answer is printed, which amounts merely to a blank denial on matters of fact, and a plea of "not guilty" on matters of opinion. Thus the article denies that the management has failed to secure the support of the district; but it quite omits to explain the falling-off of revenue, and indeed does not attempt to do so. It asserts that the hospital management is more popular than ever it was, and "has the whole-hearted support of the people of Portsmouth." Yet the revenues are decreasing! an inconvenient fact glossed over in a discreet silence. Then, too, we stated that the policy of the management has been narrow in some respects. To this the reply is that it is "altogether democratic"; and the wording of the sentence shows that this is intended as a full and complete denial of the charge. But surely a democracy can perpetrate acts as narrow as an oligarchy? It does not affect the issue in the least that the policy is democratic. Lastly, the article defends the recent proposals concerning the status of surgeons, which were fortunately defeated. It assumes that the prevailing restrictions (concerning dispensing and so forth) amount to a boycott of the "poor man's doctor"; but it does not answer any single one of the arguments we adduced, and it fails to advance any other argument whatever in favour of the change. It is therefore unnecessary to bring forward again the contentions of our leading article of a fortnight ago; but it is perhaps permissible to express the hope that the editor of the *Hampshire Telegraph* will never require to learn by personal experience the difference between the operative technique of an expert surgeon and that of one whose opportunities are limited by the demands of a heavy working-class or dispensary practice.

### NEW CHAPLAIN APPOINTED AT HEREFORD.

The Rev. H. G. Sargent has been appointed honorary chaplain to the Victoria Eye and Ear Hospital, Hereford. He succeeds the Rev. C. A. Treherne, who is stated to have resigned the post on account of the increasing claims of parochial work. At the present time, too, the responsibility of the hospital chaplain is an increasing one, for not only does he have his duties towards the patients, but on his shoulders is thrown a share in the work of reviving the appeal of Hospital Sunday, which financially and from the hospital's point of view has been sadly going downhill of recent years. Every hospital chaplain to-day has a fine opportunity of recalling the early spirit of this great movement. The work is there if only the men can be found to devote their energies to it as officials responsible, like the other members of the staff, for the hospital's well-being.

### A COTTAGE HOSPITAL'S MEDICAL STAFF.

It was recently asked at the annual meeting of the Fleet Cottage Hospital why a member of a recently formed Friendly Society which, we believe, subscribes to this institution, should not have the benefit of the Society's medical officer. Mr. Lindsay Johnston, J.P., who was in the chair, said that it had been decided that "it would be certainly wiser to adhere to the number of the medical officers on the hospital staff." Unfortunately the questioner did not press his question. It would have been well worth while to do so, for experience teaches that every patient served by a cottage hospital should have the opportunity of being attended by his own medical attendant. As we pointed out only last week in connection with a similar situation at Wood Green Cottage Hospital, "the right way is to allow all doctors to attend their own patients" if unpopularity, want of support, and disputes are to be avoided.

### THE LEAGUE OF ROSES IN NORTH LONDON.

Among the points of importance in the past year's work of the Great Northern Central Hospital, as brought out at the annual meeting, are the reduction of the cost per bed to £98 16s.—that is to say, a decrease of £6 10s. per bed—and the success of the League of Roses, which the chairman, Mr. James Liddon Smithett, stated to have collected a considerable amount in small sums in North London. When this League was started we drew attention to its organisation and scope, and it is therefore very gratifying to us to record the success which has attended its efforts. This success is only another proof that the methods adopted by the League of Mercy from its foundation, by which a growing body of supporters is made responsible for the collection of small sums, are infinitely fruitful and allow of modification and imitation on a smaller scale by different institutions. Not the least of its achievements has been to inspire efforts of this kind, the success of which we chronicle here for the benefit of other hospitals which are considering how best to extend the support they receive from their immediate neighbourhood.

### ONE SIDE OF AFTER-CARE AT THE LONDON.

The "Marie Celeste" Samaritan Society in connection with the London Hospital is one of those agencies which play an important part in the constructive side of hospital work, a side which every year is of growing importance. At the annual meeting this week it transpired that the Society provides surgical appliances, arranges for patients to enter the fifty-five convalescent homes which are open to it, assists families the bread-winner of which is in hospital, and supplies milk to the most destitute of the women delivered by the midwifery staff of the hospital. It was remarked at the meeting that no less than one-third of the cost of surgical appliances was collected from the patients themselves, and almost an equal amount was raised for the expenses of the patients' convalescence. To any member of the public who has an inkling of hospital work, its reconstructive side has surely a greater appeal than any other. It is the most hopeful part of the work, and it requires the least imagination and knowledge to realise that after-care in all its forms is the necessary preliminary towards making hospitals the true factory of health, which they cannot become if their work for want of money is to be confined to the mere admission of patients whose illness requires in-patient treatment. On these grounds we sincerely hope that energetic support may be forthcoming for the "Marie Celeste" Samaritan Society this year.

### LORD WANDSWORTH'S BEQUESTS: A SUGGESTION.

The enormous sum—about one million sterling—bequeathed by the late Lord Wandsworth to found an orphanage is the outstanding feature of charitable benevolence this week. But there are also features in the will which are of more direct interest to hospital workers. Thus the Bolingbroke Hospital gets £5,000; the London and St. George's £1,000 each; and the Cancer Hospital £500. Besides this, £10,000 is to be allotted for medical research as Sir William Bennett shall decide is best. This surgeon is the senior member of the surgical staff at the Dreadnought Hospital and is consulting surgeon to St. George's. The responsibility thus placed upon him is a heavy one, but Sir William Bennett is a man of affairs so thoroughly *au fait* with medical needs that his disposition of the sum is sure to be a wise one. In view of the large endowments for research which recent years have provided (the Beit Fellowships, for instance), it is a pity that something more should not be done for medical education; and any scheme which would enable this endowment to combine such a purpose with that expressed by the testator would be of especial value.

### THE DISPENSARY KEYS.

We publish this week an interesting letter from Mr. W. B. Fitch, dispenser to St. Bartholomew's Hospital, Rochester, asking "whether it is customary or desirable that the matron of a hospital should have the keys of the dispensary." He refers to a communication signed by the house surgeon and the house physician, in which they state that it is "absurd that any unqualified person, even the matron, should have access to drugs in the dispensary." We believe that the good-will which, as a rule, prevails between the members of hospital staffs is generally sufficient to make the answer to Mr. Fitch's question depend upon circumstances, and that in consequence the practice varies at different institutions. What is the experience of our readers on the point?

### PORTSMOUTH GUARDIANS AND THE INFIRMARY.

The extension of the Workhouse Infirmary continues to cause heart-searchings to the Portsmouth Board of Guardians. It will be remembered that, soon after the extensions were begun, serious differences involving extra cost appeared between the Local Government Board's plans and specifications, and those signed at the contract, and that errors were discovered in the bills of quantities. Thereupon the Board engaged experts to report on specific questions dealing with these matters. Apparently there is a diversity of opinion between the advisers called in, and it was therefore suggested that the whole of the reports should be sent to the Local Government Board with a request for advice. An amendment, put forward by Mr. Groves, was eventually carried that the experts should be summoned to explain the various points before the Local Government Board was approached in the matter. This, no doubt, will give an opportunity for full explanations to be arrived at, for the method of sending on the reports without such explanation would look as if the Guardians desired to attempt to pass on their responsibility in the matter.

### A NEW POOR-LAW MEDICAL APPOINTMENT.

The new medical officer to the Dearnley Workhouse, Rochdale, is Mr. Joseph Corker, M.B., who already has had considerable experience of Poor Law work. Mr. Corker has previously held appointments at Chorley and Warrington, and at one time was house surgeon at Rawcliffe Hospital. He has been for two years assistant medical officer at West Derby Union Workhouse, Liverpool. There were fifty-two applicants for the above post.

### THE ALMONER IN BIRMINGHAM.

In the report of the Queen's Hospital, Birmingham, one or two points deserve special comment. Amongst these is the question of the almoner. The appointment to this post was made just previous to the last year's report. About 260 cases are stated to have passed through her hands, and we learn that "the actual work done has been of great benefit to the patients." The following comment then occurs: "There is no reason to suppose that any considerable financial saving has been made to the hospital, and the question whether the work of the almoner should be continued and the cost of it added to the standing charges of the hospital has been deferred until a further report of the work has been received." As, however, "several members of the Committee have subscribed the amount of the almoner's salary," the experiment has not increased the annual expenditure. This view of the almoner's work is, of course, an important one, but it is becoming increasingly recognised that its constructive side is the justification for its existence, and this view, rather than the hope of decreasing hospital expenditure, has led to the growing number of almoners which has been so marked a feature of recent years.

### A QUESTION FOR THE NEWCASTLE SUNDAY FUND.

Dr. A. S. Percival, addressing the governors of the Newcastle Eye Infirmary recently, inquired why the Hospital Sunday Fund contributed £57 to this institution in 1897, when last year it contributed only £18, though the patients admitted have doubled in the interval. He understood that the total received by the Sunday Fund had not decreased in the meantime. We are glad to learn that a committee of the Sunday Fund is inquiring into the system of grants, so the matter may be left for the present, though it will be instructive to learn why a reduction in the grant to the Eye Infirmary has been made.

### A DOCTOR ON HOSPITAL MANAGEMENT.

Dr. T. W. Thursfield, who last year was appointed to the consulting staff of the Warneford, Leamington and South Warwickshire Hospital, has been making some important suggestions for its improved management. He has urged that no man should be chairman of more than one committee, that to secure a continuous flow of new workers each committee should elect its own chairman, and that one-third of every committee should retire each year and be ineligible for re-election till a further twelve months shall have passed. Moreover, he has succeeded in persuading the committee to appoint an honorary treasurer in place of the cashiers of the hospital's bank, who have been the nominal holders of this office hitherto. Mr. A. Cay has been appointed, and it is hoped that, in Dr. Thursfield's phrase, he may be successful in "raising the wind." Another suggestion was for the formation of a Ladies' Visiting and Samaritan Committee. These suggestions, we understand, have been accepted, at any rate in principle, and, apart from their intrinsic value, are worth quoting as showing how inventive on occasion a medical man can show himself to be when the question of supplying the management with new ideas is brought forward.

### ANOTHER RECRUIT TO THE UNIFORM SYSTEM.

The Chairman of the Worcester General Infirmary, Mr. Francis J. A. Wood, pointed out at the annual meeting of the Governors that the balance-sheet this year is presented, for the first time, on the Uniform System of Hospital Accounts, which made a detailed comparison with the statements of previous years impossible. Passing on to other subjects, he hoped the memorial annexe would soon be completed and begin its era of useful work. Everybody knew that the present accommodation for out-patients and children was seriously defective. It was hoped that by the time the annexe was opened, the Hon. Treasurer would have received enough to wipe out the debt. The Chairman also moved that the trustees be authorised to sell out of funded property sufficient to produce a sum not exceeding £2,500 to meet current expenses. He said he did so with regret, though the resolution was not so alarming as might appear, as they did not want to sell anything at present. He explained that, as the Governors would not meet for a year, they wished to have authority to sell if necessary, though it was hoped the need would not arise. The resolution was carried. As regards the Uniform System of Accounts, the transition to it is always a delicate matter. It will be recalled that we published an article last August describing how the East Suffolk and Ipswich Hospital effected the change.

### ISOLATION PATIENTS ON THE TELEPHONE.

At the Guernsey Fever Isolation Hospital, according to a pamphlet on the telephone system of the Channel Islands which has reached us, an elaborate telephone system is installed. By means of a fixed metal spring at the bedside of each patient, to which a telephone may be attached by inserting a plug in the wall, any person, if the patient's condition permits, may be put into direct communication with a relative or friend, who can talk from the bed to which he or she is confined. As a telephone message costs only one penny in the island, it is a privilege which every one can afford to enjoy. It would be curious to know in what percentage of cases the privilege is acted upon, for in the unfortunate event of an epidemic, the administration of the hospital might suffer inconvenience from the constant inquiry from the hospital exchange to the medical superintendent or ward sister concerning the fitness or otherwise of every patient in the ward to "answer a call." Isolation, if slightly complicated administratively, certainly loses its worst terrors from a patient's point of view, who, with a telephone at his bedside, can look forward to some moments of distraction during perhaps a tediously long stay in the wards.

### THE DOUBLE FUNCTION OF A WORKERS' COMMITTEE.

The formation of a workmen's executive committee in connection with the Victoria Hospital, Accrington, has been much applauded by various officials of the institution. As its chairman, Mr. R. Horrocks, pointed out, the people of the town are willing to give to the hospital, but have no system by which the money can be collected. It is hoped that this committee may increase the income by £1,000. We share that hope, and believe that this result can be secured if every factory and workshop is induced to adopt the system. But, apart from its financial aspect, there is another which is of vital importance and which has always to be considered where workmen's committees are formed. These committees bring the hospital into close touch with its town, and by increasing the number of individual workers who press the claims of the hospital and are experienced in its benefits, local public opinion is kept enlightened as to the hospital's work. There is exceptional need for this enlightenment at Accrington. More than one speaker at the recent annual meeting referred to the "disgraceful rumours" which had been circulated about the hospital, and another said that since he had been on the committee no complaint had ever been found to have any real foundation. Complaints, however, still appear to have been made. This, in all probability, merely means that the operatives, for whom the institution was mainly built, are out of touch with the hospital and its life. A workmen's committee should remedy this completely, for until the hospital can collect several workers as keen as Mr. Horrocks and others, it will never be firmly established, as it ought to be, in the affection of the district.

### THE BITTER CRY OF THE HOUSE-PAINTER.

A deputation from the Cork Painters' Society waited upon the committee of the North Infirmary, Cork, last week, alleging the "very serious grievance" that the painting in the institution was done by the inmates under the supervision of the nuns. "This sort of thing caused at present a great deal of want of employment to painters." The deputation further stated that it had waited on other boards with reference to the matter and had received a very favourable reception. It was decided to refer the matter to the Finance Committee, so that the practice of the sisters might be ascertained. Local feeling apparently is on the side of the painters, but their claim is a large one, how large may be judged from the remarks of one speaker, who said, with evident astonishment, "Some private property owners in Cork are painting their property themselves. I actually saw a man painting his own door!" The sense of outrage in this remark seems to us a trifle exaggerated.

### A CORRIDOR AS AN ANÆSTHETISING ROOM.

The Barnsley British Co-operative Society is giving a new theatre unit to the Barnsley Becket Hospital, and had the satisfaction of seeing the hospital's president, Mr. John Elliott, unveil a tablet recording the Society's gift. On this was written: "This operating theatre, with its surgical appliances and adjoining room, is the gift of the members of the Barnsley British Co-operative Society, Limited, to commemorate the jubilee 1862-1912." The theatre is estimated to cost over £1,000, and a bed has been endowed in the hospital, also by the Society, for half that sum. Mr. Elliott dwelt on the improved conditions for operations which a proper unit affords by providing an anæsthetising room. Previously, he stated, either the theatre itself, or the corridor, where people were passing, had been used for this purpose, a fact enough to show how badly an up-to-date theatre was needed at the hospital.

### AN ELABORATE OUT-PATIENT DEPARTMENT.

The report of the Royal Hospital for Diseases of the Chest in City Road, which was presented on Tuesday last, had two matters of special importance to refer to in the reconstruction of its out-patient department and in the proposal of the local health authorities of Shoreditch and Finsbury to establish a joint tuberculosis dispensary for the two districts. It has been decided to give the tuberculosis dispensary a place in the new out-patient buildings. It is argued that the increased work and usefulness for which the preventive methods of the dispensary will be responsible will increase the hospital's popularity and be reflected in an improved subscription list. The new out-patient department thus assumes an increased significance, and, as a lecture hall, a pathological museum and a research laboratory were imposed as conditions by the anonymous donor for his gift of £6,000, the new department will be in some respects unique and its completion is being awaited with unusual interest.

### JOINT BOARDS AND PHTHISICAL CASES.

Another side of the Government's proposed measures for the treatment of phthisical cases is seen in the reported suggestion of the Local Government Board that the Evesham Joint Hospital Board should relinquish its powers for dealing with the matter. The chairman said that the county was the authority best able to deal with cases, and it was agreed to apply for the amendment of the existing provisional order, so that the constituent authorities of joint hospital boards might be generally free to deal with the matter. Until it is more definitely known what machinery and means the Government's proposals are intended to employ, any re-arrangement of existing powers seems likely only to cause unnecessary trouble later on.

### A COLLIERY PRACTITIONER ATTACKED.

It is stated that Dr. Wills, of Burryport, who holds an appointment connected with a local colliery, was assaulted on his return home last Saturday evening. Seven or eight men rushed on him, but luckily Dr. Wills was returning from drill with a Territorial regiment, and drawing a revolver from his pocket he struck one of his assailants with the butt. On calling for assistance the men made off, leaving Dr. Wills, however, bruised on the legs and body. It is not clear at the time of writing what motive his assailants had. Could it be, in view of certain leaflets that have been distributed lately, that he was mistaken for one of the military, the presence of which, in any form apparently, is much resented in any of the disturbed areas? In any case it is small encouragement for a busy practitioner to join the Territorials if he is to run the risk of this sort of thing.

### WOMEN DOCTORS IN RESIDENT POSTS.

"Male doctors only" is the restriction which the Southampton Guardians have now added to their advertisement for a resident medical officer at the infirmary. In answer to a previous advertisement applications had been received only from women practitioners, and then only two candidates presented themselves. It was added that the qualifications of the women doctors were highly satisfactory, but, in spite of the efforts of two lady guardians which gained a large measure of support, it was decided not to proceed to an interview. The success of women doctors as residents in Poor Law infirmaries is sufficiently untried to account for the above action, though it is possible that in time, when the infirmaries are properly separated from the workhouses, such appointments will find an increase of favour.

### A MODEL HOSPITAL REPORT.

The annual report of the Leicester Infirmary is one of the few yearly compilations on institutional routine and management which compel attention from the interest and attractiveness with which care and brains invest them year by year. Its two hundred and fifty odd pages form a bulky pamphlet, made as readable as print, paper, and a liberal interspersion of illustrations can secure. A map is also added showing the parishes from which in-patients have been received. To complete the reconstruction scheme the report states that "a small isolation hospital of some ten or twelve beds is urgently needed," and that "the reconstruction of the remaining wing, containing accident and

St. Luke's wards," is desirable. Were these alterations, together with the reorganisation of the kitchen department, carried out, we understand that the last sections of the reconstruction scheme resolved on about ten years ago would be completed. By the way, we notice that the old rule that "patients should bring with them knife, fork, and spoon, two towels, soap, slippers, a tooth-brush, and change of linen" still survives. The arguments against this requirement are to be found in "Burdett's Hospitals and Charities" for 1893, in which chapter six is devoted to comparing the institutions which favour with those which reject it. There it is urged that "discipline, cleanliness, and due regard to the circumstances of the poor" demand its abolition, while it is remarked that "Strange to say, the demands made on patients for supplies are greater at those hospitals where the cost per bed is higher than at the Scotch hospitals, where the cost of an unoccupied bed is relatively small." After twenty years, however, a complete abolition of the practice is yet to seek.

### A SUCCESSFUL ANNIVERSARY AT BRISTOL.

Exactly a year ago the Lord Mayor of Bristol issued an appeal for £45,000, on behalf of the committee of the Bristol General Hospital. Of this sum £42,850 has been received, a very satisfactory result considering the difficulties and disturbances with which hospital work has been hampered since March 1911. We understand that a beginning has been made by clearing the site on which the new wing will be built. That wing, it should be recalled, will include the female medical and maternity wards, with their southward facing balconies, while an important part of the enlargement scheme is to be the new dental department which will take the place of the present department some distance away. A roof garden for convalescent patients is contemplated, and the resident medical staff will have additional accommodation. Bristol has often proved itself a loyal supporter of the voluntary system, and the quick response to this appeal shows that the old spirit is still alive, in spite of recent legislation.

### THE SOUTH INFIRMARY, CORK.

An advance copy of the annual report of the South Infirmary, Cork, discloses the remarkable fact that much of it is devoted to an appeal for support on account of the Insurance Act, notwithstanding the fact that the medical benefits section does not apply to Ireland. If so much alarm is felt in Ireland, where, we presume, the public cannot be cajoled into the belief that the hospitals can in any way benefit by the provisions of the Act, there is no need to emphasise the alarm in England, cloaked though that may be at present under the hope that things may work out better than they promise to do. This report shows that an Irish hospital is not likely to have any illusion as to the effect on voluntary contributions which compulsory insurance will have. The report, we may add, is more attractive than those of many English hospitals; illustrations are scattered through it, and it should attract attention and claim the interest of all into whose hands it may fall.

### "THE HOSPITAL" PENSION.

At the annual general meeting of the Newsvendors' Benevolent and Provident Institution, held on March 15, the Committee gave notice that Charles Skeats has been elected to be the recipient of The Hospital Pension for the year 1912. The President was requested to convey to the proprietors of The Hospital the hearty thanks of the members of the institution for the generous assistance afforded by The Hospital Pension.

### THIS WEEK'S DRUG MARKET.

There are no signs of improvement in the drug and chemical markets, and no improvement can be expected until the coal strike is over. For the time of the year business is unusually quiet and there can be no doubt that the dulness is due, directly and indirectly, to the disturbances in the coal trade. From the mining and other districts where men are out of employment and money has ceased to circulate freely, wholesale druggists are naturally receiving smaller orders, while those manufacturers of chemicals and galenical preparations who are sparingly using their supplies of fuel are not in any haste to purchase crude drugs and other raw materials. The falling off in demand, however, has not had a depressing effect on prices, which are for the most part well maintained. The alterations in the value of drugs have in fact been few, and in hardly any instance is it possible to trace any rise or fall in price to the coal strike. Up to the present makers of corrosive sublimate, calomel, etc., have announced no alteration in their quotations for these products as a result of the rise in value of quicksilver. Cod-liver oil has declined in value still further, and present quotations are between one-half and two-thirds the prices quoted at the beginning of the year; it is quite probable that a further reduction will take place, but there is not now so much inducement to refrain from making purchases as there was a week or two ago. Santonin is unaltered at the recent advance; the excessively high price which is quoted for this drug has had the effect of encouraging adulteration, and samples have been offered which are adulterated with acetanilide; under the circumstances it would be well if buyers obtained a guarantee that their purchases are unadulterated. Opium still shows a tendency to decline in value, as also does morphine, but there are at present no signs that any substantial reduction is pending. Belladonna root is rather cheaper. Cloves are dearer, as also is chiretta.

### TO OUR READERS.

Contributions are specially invited from any of our readers to these columns. They should deal with topical subjects and news. They must be authenticated for the information of the Editor only. The minimum payment if published is 5s. There is no hard-and-fast rule as to space, but notes of about twenty lines in length are preferred.

